# Endothelial Expression of TGFβ Type II Receptor Is Required to Maintain Vascular Integrity during Postnatal Development of the Central Nervous System

**DOI:** 10.1371/journal.pone.0039336

**Published:** 2012-06-26

**Authors:** Kathleen R. Allinson, Hye Shin Lee, Marcus Fruttiger, Joseph McCarty, Helen M. Arthur

**Affiliations:** 1 Institute of Genetic Medicine, Newcastle University, Newcastle upon Tyne, United Kingdom; 2 University of Texas MD Anderson Cancer Center, Houston, Texas, United States of America; 3 UCL Institute of Ophthalmology, University College, London, United Kingdom; Children's Hospital Boston, United States of America

## Abstract

TGFβ signalling in endothelial cells is important for angiogenesis in early embryonic development, but little is known about its role in early postnatal life. To address this we used a tamoxifen inducible Cre-LoxP strategy in neonatal mice to deplete the TypeII TGFβ receptor (Tgfbr2) specifically in endothelial cells. This resulted in multiple micro-haemorrhages, and glomeruloid-like vascular tufts throughout the cerebral cortices and hypothalamus of the brain as well as in retinal tissues. A detailed examination of the retinal defects in these mutants revealed that endothelial adherens and tight junctions were in place, pericytes were recruited and there was no failure of vascular smooth muscle differentiation. However, the deeper retinal plexus failed to form in these mutants and the angiogenic sprouts stalled in their progress towards the inner nuclear layer. Instead the leading endothelial cells formed glomerular tufts with associated smooth muscle cells. This evidence suggests that TGFβ signalling is not required for vessel maturation, but is essential for the organised migration of endothelial cells as they begin to enter the deeper layers of the retina. Thus, TGFβ signalling is essential in vascular endothelial cells for maintaining vascular integrity at the angiogenic front as it migrates into developing neural tissues in early postnatal life.

## Introduction

Developing neural blood vessels need to maintain an appropriate interaction between endothelial cells and neural cells for successful angiogenesis. During brain development, pericytes are recruited to migrating endothelial cells (ECs) at an early stage of blood vessel formation to initiate blood brain barrier (BBB) properties. Astrocytes are subsequently recruited, approximately a week later in murine development [1]. Thereafter, pericytes play a critical role in integrating endothelial and astrocyte interactions to maintain the BBB during cerebral development and into adult life [1], [2], [3], [4]. Failures to form appropriate inter-cellular contacts can lead to vascular haemorrhage, which has severe consequences. For example, cerebral haemorrhage, seen in approximately one fifth of premature infants in developed countries, may result in permanent deficiencies in cognitive and motor functions [5], [6].

Similar EC-pericyte-astrocyte interactions occur in the retinal vessels to form the blood-retinal barrier (BRB) [7]. During development, retinal astrocytes first migrate from the optic nerve across the surface of the retina, and ECs migrate across the astrocyte layer and recruit pericytes to form an organised vascular plexus that grows by sprouting angiogenesis. After reaching the periphery of the plexus (normally at day 7 in the mouse retina), further vessels branch primarily from the veins and grow down along Muller cell processes into the neural plexus to form two further ‘deeper’ vascular networks on either side of the inner nuclear layer. Unlike the primary plexus, the two deeper layers of vessels initially form in an astrocyte independent manner in a similar way to cerebral vessels, and astrocytes are recruited after pericyte recruitment [7].

Neural blood vessels develop primarily by sprouting angiogenesis which involves several extracellular cues and intracellular signalling pathways [8]. For example, VEGF is released by hypoxic tissues and directs migration and proliferation of ECs. Differentiation of leading tip cells and adjacent stalk cells are maintained through a dynamic Delta/Notch signalling program [9]. In addition, PDGF signalling is critical for recruitment of pericytes to the ECs that lie behind the leading edge of the plexus [10]. However, certain signalling pathways appear to be particularly important for angiogenesis in developing neural tissues. For example, Wnt7a and Wnt7b ligands released by the neuroepithelium activate canonical Wnt signalling in migrating ECs and defects in this pathway leads to vascular haemorrhage and disrupted neural angiogenesis [11], [12], [13].

**Figure 1:**
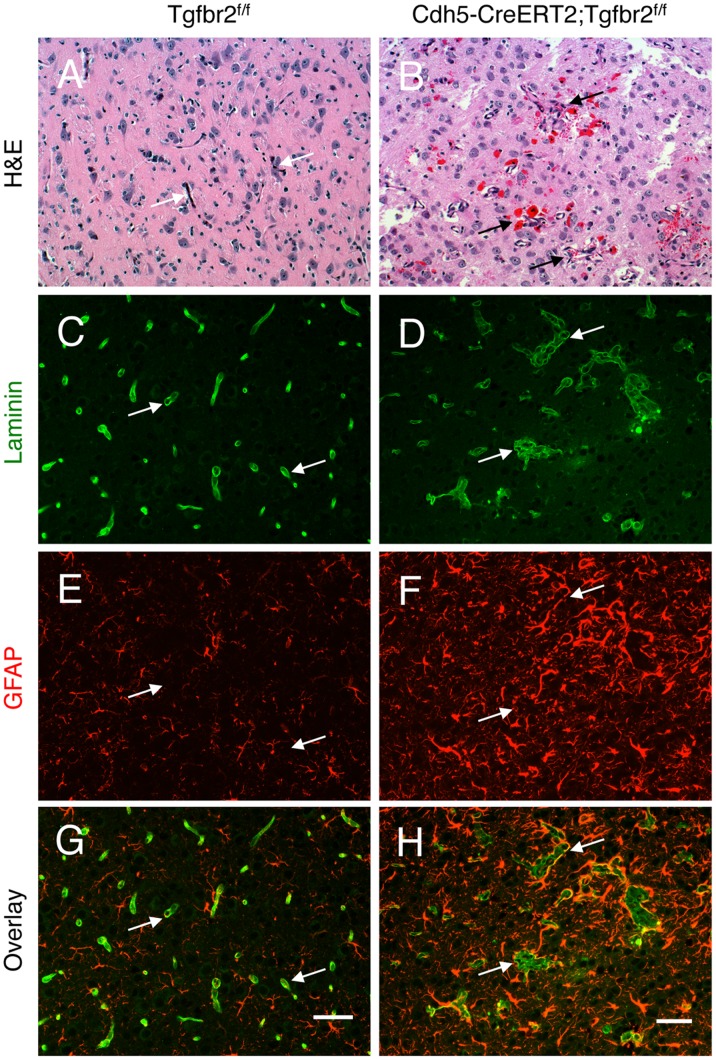
Loss of Tgfbr2 in neonatal endothelial cells leads to cerebral vascular pathologies and intracerebral haemorrhage. Coronal sections through cerebral cortices of P14 control (A) and Tgfbr2–iKO^e^ mice (B) were stained with H&E. Note the abnormal blood vessel morphologies and microhaemorrhage in Tgfbr2 mutant brains. (C-H); Coronal sections through cerebral cortices of P14 control (C, E, G) and Tgfbr2 conditional mutant mice (D, F, H) were double immunofluorescently labelled with anti-laminin (C, D) and anti-GFAP (E, F) to visualize vascular basement membranes and astrocytes, respectively. Note the cerebral blood vessels with glomeruloid-like tufts (D) as well as robust perivascular astrogliosis (F) in Tgfbr2-iKO^e^ mutant sections. A total of 5 mutant and 4 control mice were examined for brain pathologies. Scale bars: 50 µm.

Recent work has also pointed towards the importance of TGFβ signalling for angiogenesis of developing neural tissue. Combination of mutations in *Tgfb1* and *Tgfb3* genes leads to loss of cerebral vascular integrity and haemorrhage [14], [15]. Similar phenotypes result from the absence of the integrins αvβ8 expressed by glia and required for activation of TGFβ ligands [16], [17], [18], [19]. We have previously shown that endothelial cell-specific loss of the TGFβ type II receptor (Tgfbr2) in embryos at E11.5 (using a tamoxifen inducible Cre approach) resulted in cerebral haemorrhage in the forebrain and embryonic lethality at E15.5 [20]. Recently, a similar phenotype was reported in another endothelial specific *Tgfbr2* null mouse where vascular defects and haemorrhage were also localised within the intraneural tissues of the developing forebrain [21]. Using the tamoxifen inducible Cre approach, we now demonstrate that vascular haemorrhage also occurs in retinal and brain tissues when Tgfbr2 is depleted postnatally in ECs, supporting a continued role for Tgfbr2 in neural ECs following birth. We have focussed our analysis on the retinal vasculature, which has allowed a more detailed investigation of the disturbed cellular events.

**Figure 2:**
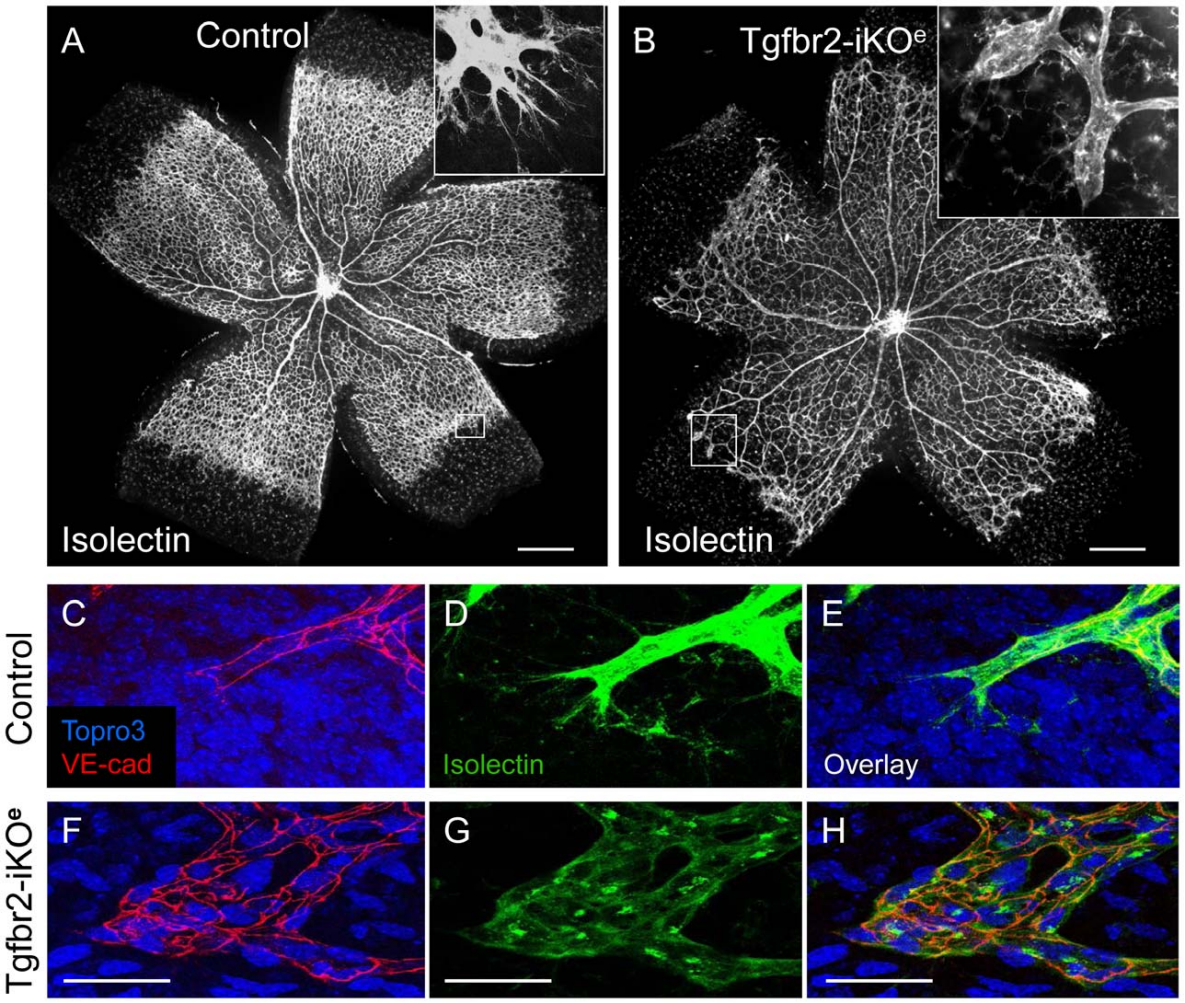
Tgfbr2-iKO mutants show vascular abnormalities in the postnatal retina. Isolectin stained retinal preparations at postnatal day (P)7 show normal vascular architecture in controls (A), but reduced branching in Tgfbr2-iKO^e^ mutants (B). Frequently, round clusters of endothelial cells (boxed area and inset, B) are seen at the leading edge of the vascular plexus in mutants, at the positions where tip cells normally occur in controls (boxed area and inset, A). High power views of whole mount P9 retinal preparations stained with VE-cadherin and isolectin show the endothelial footprint of a normal tip cell (C,D,E) compared with that of the endothelial cell clusters in the Tgfbr2-iKO^e^ mutant (F,G,H). Scale bars: 500 µm A,B; 50 µm, C–H.

## Results

Mice homozygous for the floxed *Tgfbr2* allele [22] and carrying the tamoxifen inducible *Cdh5(Pac)Cre^ERT2^* transgene [23] were treated with tamoxifen at postnatal day (P)2 and P4 to deplete the *Tgfbr2* gene specifically in ECs and generate Tgfbr2-iKO^e^ mice. Q-PCR was used to confirm knockdown of *Tgfbr2* expression in Tgfbr2-iKO^e^ retinal endothelial cells (Figure S1A). Tamoxifen treated *Tgfbr2^fl/fl^* littermates acted as controls. The morphology of the cerebral vasculature was severely disturbed in the Tgfbr2-iKO^e^ mutants. Using anti-laminin staining to reveal vascular basement membranes, we found tortuous blood vessels displaying glomeruloid-like vascular tufts throughout the cerebral cortices as well as in other brain regions, including the hypothalamus. Significant intracerebral micro-haemorrhages, visible as extravasated red blood cells, were also detected at P14 (Figure 1). These blood vessel pathologies were associated with an abnormal increase in the number of GFAP-expressing reactive astrocytes. However, not all brain regions showed vascular defects, suggesting heterogeneity in Tgfbr2 gene expression and/or mosaic patterns of Cre expression and tamoxifen-inducible activation.

**Figure 3:**
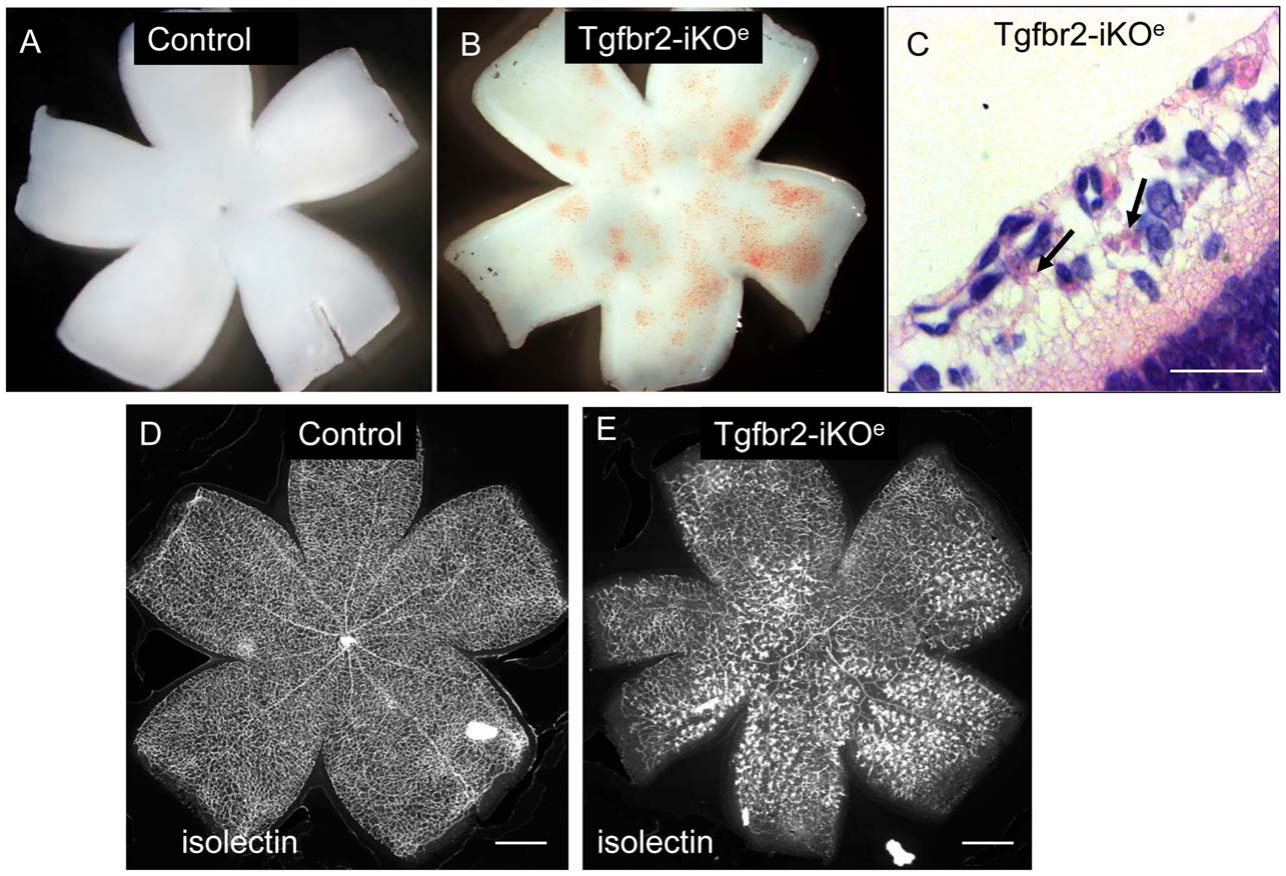
Retinal haemorrhage in Tgfbr2-iKO^e^ neonates upon development of the secondary vascular plexus. Stereo-images of freshly dissected whole mount P9 retinas show multiple microhaemorrhages in the Tgfbr2-iKO^e^ that are not seen in controls (A,B). H&E stained sections of Tgfbr2-iKOe retina show regions of microhaemorrhage within the retinal tissue (arrows,C). Wholemount view of isolectin stained P14 retinas show multiple glomerular tufts (seen as intensely stained clumps of ECs) in the Tgfbr2-iKO^e^ mutants that are not seen in controls (D,E). This phenotype was seen in over 100 mutant retinas from P8 to P28. Scale bars: 500 µm, C,D; 20 µm C.

**Figure 4:**
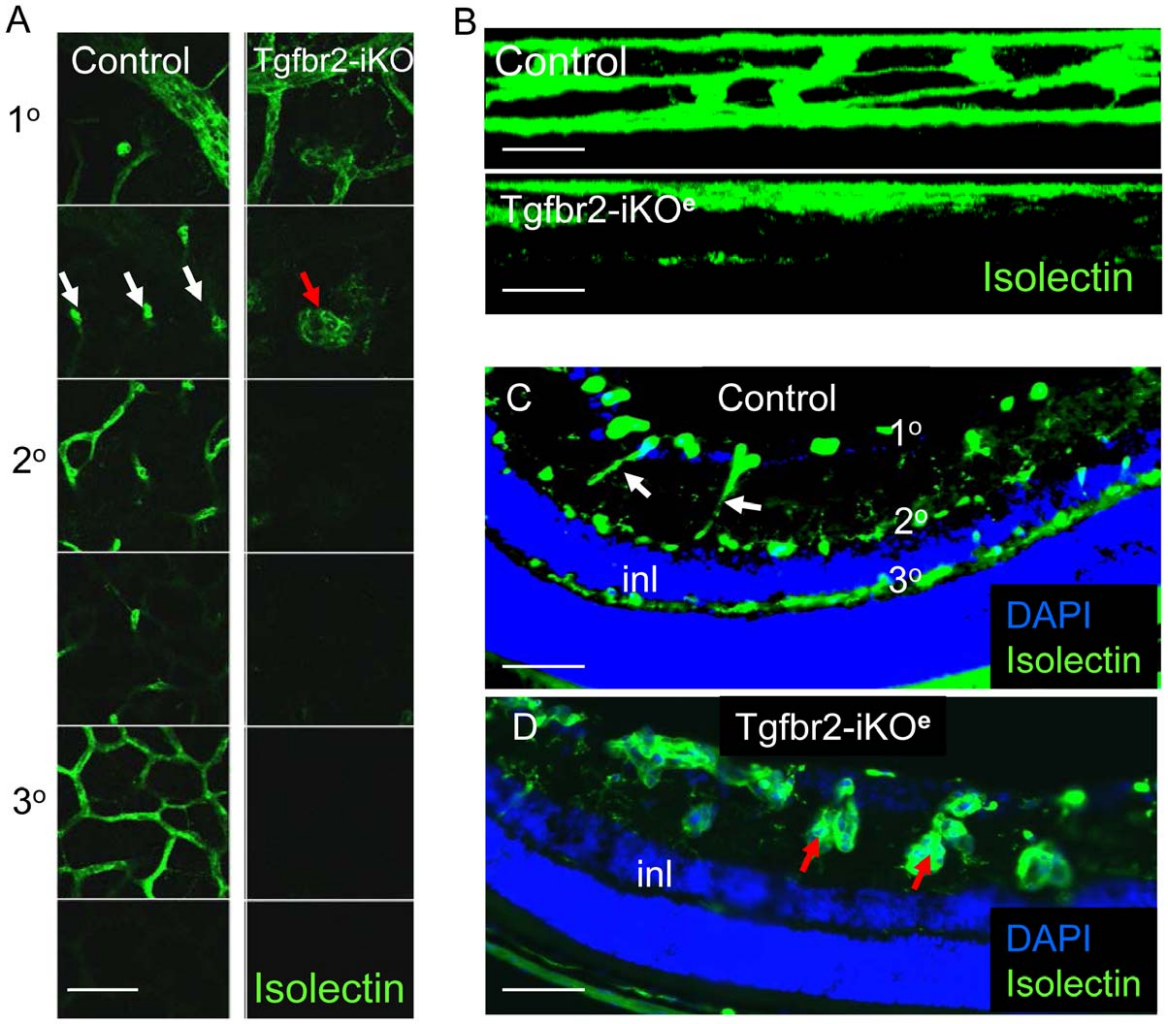
Formation of the deeper vascular network is severely impaired in Tgfbr2-iKO^e^ mutant retinas. Confocal slices of isolectin stained retinas from P14 neonates shows presence of primary, secondary and tertiary networks in controls, but only the primary plexus in Tgfbr2-iKO^e^ mutants. Z-slices showing surface view is shown in panel A whereas side view of vascular plexus is shown in B. Section through a control retina at P21shows the normal organisation of the primary, secondary and tertiary vascular plexus with respect to the surface of the retina and inner nuclear layer (inl). The interconnecting vessels between the primary and secondary plexus are regular small capillaries in controls, indicated by white arrows in Figures A and C. In contrast, this part of the deeper plexus in the Tgfbr2-iKO^e^ retinas contains glomerular tufts (red arrows, A and D). Scale bars: 50 µm A,C,D; 20 µm B.

In order to examine these neural angiogenesis defects in more detail we used the neonatal retina, where the timing and stages of development of the primary and intraneural vasculature are well characterised [7]. The primary network of astrocytes at P6 appeared similar between mutants and controls (Figure S2). However, analysis of the retinas at P7 showed that there was reduced vascular branching in the Tgfbr2-iKO^e^ mutants and a small but significant delay in vascular plexus migration compared with controls (Figure 2 A,B; Figure S1B,C). A striking feature of the mutant vascular plexus at P7 was the frequent occurrence of abnormal clusters of ECs at the periphery, where the characteristic angular and elongated tip cells are normally present. The ECs within these clusters also lacked the typical long filopodial projections that are characteristic of tip cells (Figure 2, compare D and G). Endothelial adherens junctions (revealed by VE-cadherin staining) (Figure 2,C–H), and tight junctions (Claudin 5 staining, Figure S3) were present in Tgfbr2-iKO^e^ retinal vascular ECs, suggesting EC junctions were formed in the absence of endothelial Tgfbr2.

Once the retinal vascular plexus began to migrate into the underlying neural tissue at P8, numerous microhaemorrhages developed in the Tgfbr2-iKO^e^ retinas that were not seen in controls (Figure 3A,B). The haemorrhagic areas were associated with large numbers of glomerular tufts of ECs (Figure 3C,E) that occurred most frequently near retinal veins in the primary plexus. These retinal veins are the major source of new vessels that branch into the neural tissue to form the secondary and tertiary plexus of the deeper network. The timing and location of the retinal haemorrhage appeared to be co-incident with defective migration of blood vessels into the deeper layers of the retina to form the secondary and tertiary plexuses. We therefore examined these deeper regions using confocal microscopy and found there was a failure of the ECs to form the secondary and tertiary plexus (Figure 4A and B). This was not due to a delay in vascular development, as the defect persisted even at 3 weeks of age. Analysis of tissue sections of Tgfbr2-iKO^e^ mutant retinas from P8 onwards showed that ECs had begun their migratory path into the neural tissues, but appeared to have stalled in their progress, resulting in ‘aggregates’ of disorganised ECs (Figure 4D, Figure S4,C–F). This was in stark contrast to age matched control retinas which showed regular small capillaries across the neural plexus (Figure 4C, Figure S4A,B).

**Figure 5:**
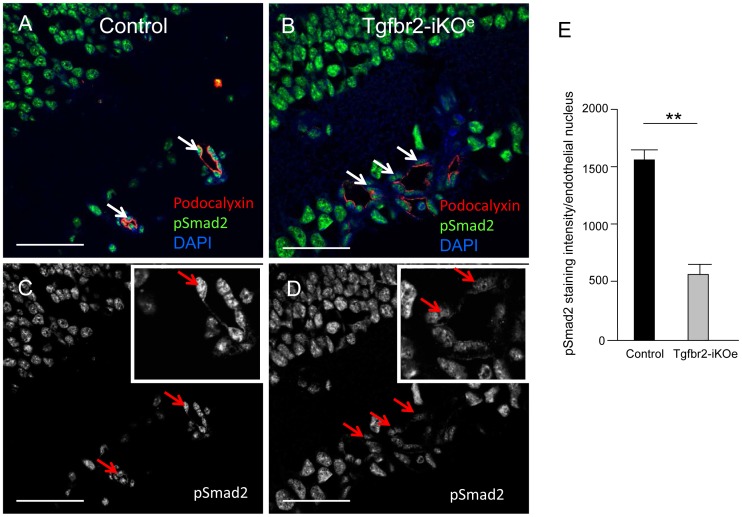
Reduced Smad2 phosphorylation in ECs in Tgfbr2-iKO^e^ retinas. Retinal sections (age P14) stained for pSmad2 (green) reveal Smad2 activation in both vascular cells and neural cells. Confocal analysis of podocalyxin staining (red) was used to identify the apical surface of endothelial cells in retinal blood vessels, and DAPI to identify the nuclei. Endothelial cells show reduced levels of pSmad2 activation in the mutant retinas (white arrows, B) compared with controls (white arrows, A). This difference can also be seen in the equivalent monochrome confocal images of pSmad2 staining in the same sections and in the digital zoom image inserts (C,D). Abbreviations: inl, inner nuclear layer. Scale bar: 50 µm. E: Quantitation of pSmad2 staining intensity using NIS-elements software was performed on 58 endothelial nuclei from 3 mutant retinas and 42 nuclei from 3 littermate controls. Endothelial cell nuclei in random fields of view were identified by podocalyxin apical staining. Statistical analysis using a student’s t-test shows a significant reduction of pSmad2 in endothelial cells of Tgfbr2-iKO^e^ mutants compared with controls. ** p<0.001.

The Cre reporter *Rosa26R* allele [24] was used to confirm the efficiency of Cre^ERT2^ activation using X-Gal staining. LacZ expression in tamoxifen treated *Rosa26R;Tgfbr2^fl/fl^;Cdh5(Pac)Cre^ERT2^* neonatal retinas confirmed the haemorrhagic glomerular tufts were comprised of ECs in which Cre^ERT2^ has been activated and continued to persist in later life (Figure S5). Here, the overlap of blue staining and glomerular tuft phenotype is important in showing co-incidence of this phenotype with regions of Cre activity.

**Figure 6:**
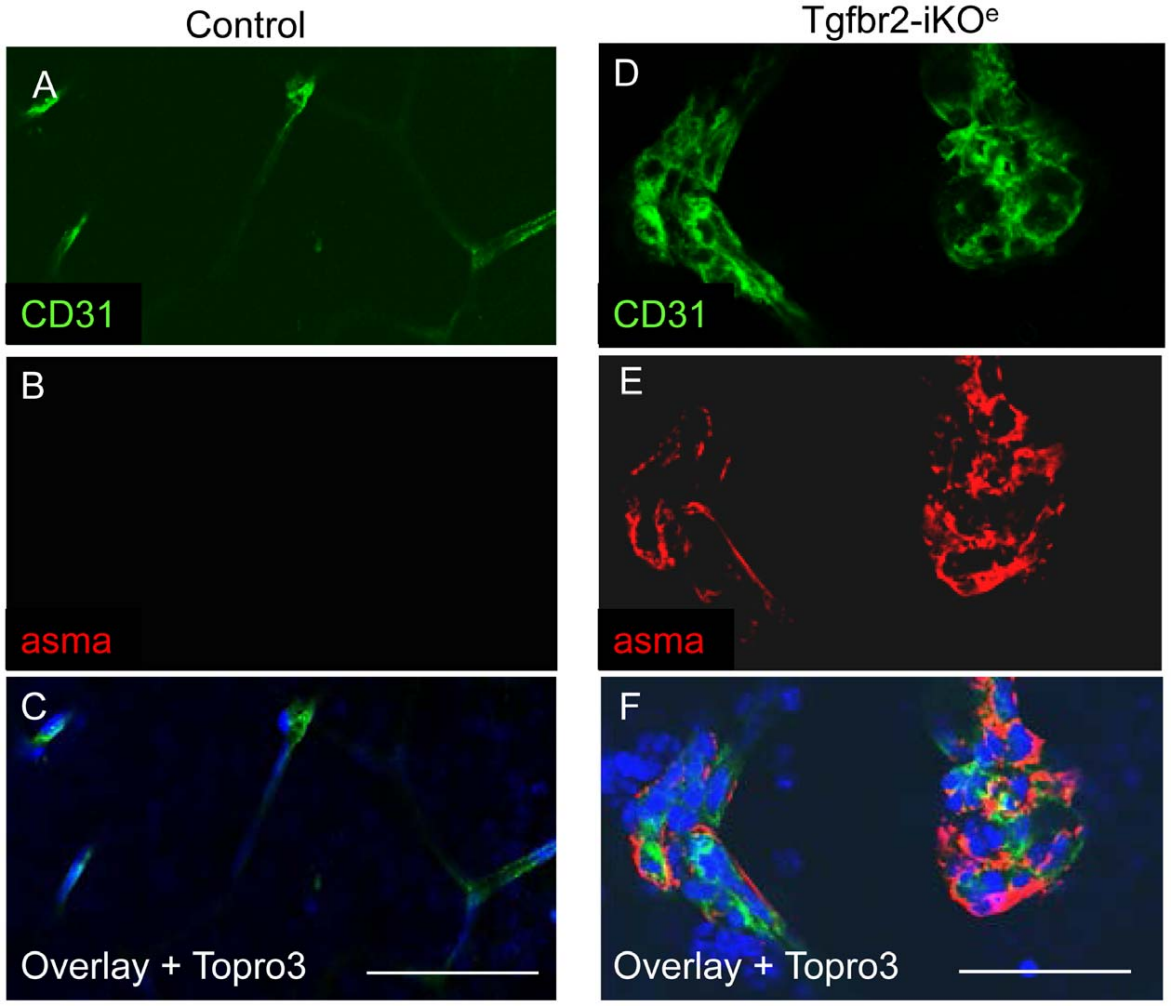
Endothelial glomerular tufts in the Tgfbr2-iKO^e^ mutants contain multiple smooth muscle cells. Immunofluorescent staining of P14 retinal paraffin sections with isolectin-alexa488 and anti-alpha smooth muscle actin (aSMA) conjugated to Cy3 show the typical non-muscularised microvessels of the control retinal plexus (arrows in A and D), whereas high numbers of vascular smooth muscle cells are associated with endothelial glomerular tufts (E,F and H). Scale bar: 50 µm.

**Figure 7:**
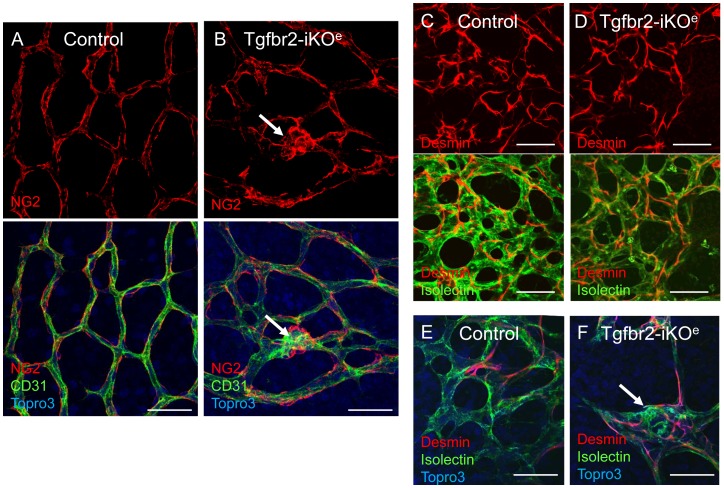
Pericytes are associated with endothelial cells in Tgfbr2-iKO mutant retinas. There were no apparent differences between mutants and controls in the organisation of pericytes on capillary retinal endothelial cells. Pericytes are identified using either NG2 (A,B) or desmin staining (C–F). Pericytes are present in the glomerular tufts in Tgfbr2-iKO^e^ mutant retinas (B, F, arrows). Similar results were seen in a total of 10 mutants and 11 controls aged between P7 and P14. Scale bar: 50 µm.

The hyaloid vasculature normally regresses as the retinal vascular plexus develops and has more or less completely disappeared by P21 in controls. In contrast, hyaloid vessels persist for several weeks in the Tgfbr2-iKO^e^ mutants and were present in all mutants examined (Figure S6). In approximately a quarter of cases, branches of the hyaloid vasculature invaded the retina and connected to retinal vessels. Persistent hyaloid vessels are a common occurrence when there is a failure in the normal development of the deeper retinal plexus [7].

Given that Tgfbr2 is required for TGFβ signalling and Smad2/3 phosphorylation, we next examined Tgfbr2-iKO^e^ retinas for changes in phospho-Smad2 (pSmad2) levels. Confocal analysis of pSmad2 staining showed there were significantly reduced levels of pSmad2 in the ECs in the Tgfbr2-iKO mutants compared with controls (p<0.001, Figure 5). TGFβ signalling is also involved in maintaining cell survival, but we were unable to detect any difference in endothelial cell apoptosis in Tgfbr2-iKO^e^ retinas compared with controls as judged by the levels of activated caspase 3 staining (not shown). To evaluate whether EC proliferation contributed to the glomerular tuft phenotype we used double staining with CD31 and BrdU and found ECs in these glomerular-like vascular structures were actively proliferating in the mutant retinas (Figure S4H).

The interaction of ECs with vascular smooth muscle cells/pericytes is critical during neural angiogenesis and TGFβ signalling from ECs has been reported to promote the maturation of vascular smooth muscle cells during vascular development [25]. Also, we have previously shown that defects in TGFβ signalling in ECs can lead to reduced vessel muscularisation in the yolk sac [26]. We therefore expected to see reduced muscularisation of vessels in Tgfbr2-iKO^e^ mutants, However, we observed no reduction in smooth muscle cells (detected by staining for alpha smooth muscle actin) in mutants compared with controls (Figure S7A–D). Surprisingly, the numbers of vascular smooth muscle cells were actually increased in Tgfbr2-iKO^e^ retinal capillaries compared with controls, a feature that was first observed at P7 (Figure S7E,F). Furthermore, the glomeruloid tufts also contained numerous vascular smooth muscle cells (Figure 6) that were proliferating in a similar way to the ECs (Figure S8). A 3D reconstruction of confocal z-slices illustrates the contrast between the non-muscularised capillary network of the primary and secondary plexus in the normal retina and the muscularised glomerular tufts that form as downward growths from the primary plexus penetrate the neural tissue (see movies S1 and S2). Thus, there was an abnormal distribution of proliferating vascular smooth muscle cells, but no evidence for a defect in smooth muscle cell differentiation. As pericytes are key to the generation of the BRB, we also used anti-desmin and anti-NG2 antibodies to examine the organisation of pericytes on developing retinal vessels. Surprisingly we observed no differences in pericyte organisation on retinal vasculature except that the endothelial glomerular tufts present in the mutants contained pericytes (Figure 7). Taken together this data suggests there was not a failure in vascular smooth muscle cell differentiation or recruitment in the absence of endothelial Tgfbr2, but rather there was a failure of progression of the angiogenic sprouts into the neural plexus resulting in an aggregate of proliferating and disorganised ECs with associated pericytes and vascular smooth muscle cells. In order to compare with another tissue undergoing active angiogenesis at the same period of development we examined the ear vasculature of mutants and controls. We confirmed that there were no abnormal glomerular tufts in this tissue in the absence of endothelial *Tgfbr2* (Figure S9), suggesting this phenotype is specific for angiogenesis of neural tissues.

## Discussion

The combination of endothelial specific depletion of Tgfbr2 and the readily accessible developing vasculature of the neonatal retina make it a valuable model for investigating the role of TGFβ signalling during angiogenesis of neural tissue. The major defect in the Tgfbr2-iKO^e^ mutants is the development of abnormal haemorrhagic glomerular tufts and failure to form the deeper vascular plexus. These defects are in sharp contrast with the retinal and phenotype in mice with endothelial specific loss of the TGFβ co-receptor endoglin [27]. Mice in which endoglin has been depleted in ECs (Eng-iKO^e^ mice) develop major arteriovenous malformations, but retain the ability to develop the deeper layers of the vascular plexus. Endoglin and ALK1, a TGFβ family type I receptor, show high affinity for BMP9 and BMP10, and are both associated with the inherited vascular disorder Hereditary Haemorrhagic telangiectasia (HHT) [28], [29], [30]. Thus, loss of Tgfbr2 does not appear to be involved in the development of arteriovenous malformations during organogenesis, in agreement with a previous report [31]. However, it remains an open question whether TGFβ signalling contributes to the formation of arteriovenous malformations and haemorrhage of the brain resulting from injury [32].

The cerebral haemorrhagic phenotype of the Tgfbr2-iKO^e^ neonates suggests there is a defective interaction between vascular cells and neural cells that specifically disrupts angiogenesis of neural tissues. The mild defects seen in the early retina suggest a slightly reduced level of migration across the astrocytes. However, the severe defects seen in the downward migrating ECs point to a greater migration defect along the Muller cells. In addition to these postnatal defects we, and others, have previously shown Tgfbr2 is also important in ECs for vascular integrity during development of the embryonic brain [20], [21]. Furthermore, endothelial specific depletion of the TGFβ type I receptor Alk5 leads to a similar cerebral haemorrhagic phenotype and embryonic lethality [21], [33], consistent with a requirement for both Tgfbr1 and Tgfbr2 proteins as a heteromeric receptor complex during TGFβ signalling in ECs.

TGFβ signalling in ECs has also been shown to be critical for close interaction with pericytes as endothelial specific loss of Smad4, a central mediator of TGFβ signalling, leads to a reduced association between pericytes and ECs resulting in intracranial haemorrhage [34]. We show here that pericytes and smooth muscle cells are recruited to the ECs in the Tgfbr2-iKO^e^ retinas, but this is insufficient to stabilise the downward migrating vessels leading to disorganised clusters of endothelial and muscle cells, and haemorrhage.

Mice deficient for αv or β8 integrin show similar vascular glomeruloid haemorrhagic malformations [35], [36]. Integrins are required to activate latent TGFβ ligands in the extracellular matrix [37] and the similarity of αv or β8 integrin null phenotypes to the phenotype described here is consistent with this role. We have shown that loss of integrins specifically in retinal neural cells leads to a similar phenotype [19] and reveal that latent TGFβ in the extracellular matrix (ECM) is activated by interaction with αvβ8 integrin allowing active TGFβ to be released for signalling in the neural endothelial cells. It has been proposed that this interaction between endothelial and neuroepithelial cells allows proper localization of glial processes around vessels [17], [18]. It is also possible that activated TGFβ in the neural ECM acts as an attractant for ECs migrating into neural tissues.

Loss of the orphan G-coupled receptor GPR124 (also known as Tem5), which is expressed in the developing vessels of the neural tissues, leads to a similar phenotype to the Tgfbr2-iKO^e^ mice described here, including the presence of vascular glomerular tufts in the forebrains of GPR124 null embryos [38]. Interestingly, ablation of Gpr124 results in perturbations of expression of genes downstream of the TGFβ pathway, suggesting that the phenotypes of Tgfbr2 mutants and Gpr124 mutants may be related.

In the early embryo, Tgfbr2 null mutations lead to failure in yolk sac angiogenesis and embryonic lethality at embryonic day (E)10.5 [39]. Endothelial specific depletion of *Tgfbr2* using Tie1-Cre or Tie2-Cre transgenes led to a very similar phenotype [40], [41], suggesting that at this early stage Tgfbr2 is required for angiogenesis in early embryonic tissues. However, our data shows that TGFβ signalling in ECs is also required for angiogenesis of neural tissues in early postnatal life. In the absence of Tgfbr2, specifically in ECs, TGFβ signalling is disrupted, as evidenced by reduced phosphorylation of Smad2, leading to the formation of disorganised haemorrhagic clusters of endothelial cells and smooth muscle cells as angiogenic sprouts begin to migrate into the neural tissue.

Tgfbr2 mutations are associated with the human disease Loeys Dietz syndrome. Some features of this disease has been modelled in mouse where Tgfbr2 has been deleted in vascular smooth muscle cells (reviewed in [42]). However, depletion of Tgfbr2 in ECs from E17.5 when the aortic wall is rapidly growing did not lead to any detectable defects in the aorta suggesting there is no endothelial contribution to this defect (data not shown). The defects reported here suggest that the EC contribution to vascular development is restricted to the central nervous system. Further work is required to understand the intimate molecular interactions between neural and endothelial cells that are required for successful intraneural angiogenesis. Improved understanding of this process is also important in the context of developing improved treatments for spontaneous cerebral microhaemorrhage in premature human infants.

## Methods

### Animals

All animal protocols were approved by the Newcastle University Ethical Review Committee and mice were maintained according to the requirements of the Animals (Scientific Procedures) Act 1986 of the UK Government. Mice in C57BL/6 background carried the previously described floxed *Tgfbr2* allele and the tamoxifen inducible *Cdh5(PAC)Cre^ERT2^* transgene [22], [23]. Subcutaneous injection of 0.5 mg Tamoxifen (dissolved in peanut oil) at postnatal day (P) 2 and P4 was given; identical doses were given to control littermates. In some cases the *Rosa26R* allele was used to monitor Cre activation [24]. Genotyping was performed by PCR as previously described [22], [27].

### Tissue Staining

Pups were humanely killed, eyes enucleated and retinas dissected before fixation in 4% (wt/vol) paraformaldehyde in PBS. Retinal tissues were stained as previously described using alexa488-conjugated isolectin B4 (Invitrogen), Cy3 conjugated antibody to alpha-smooth muscle actin (aSMA) (Sigma) and Alexa488 conjugated anti-Claudin 5 (Invitrogen); as well as primary antibodies to pSmad2 (Cell Signaling); Ve-Cadherin, Endoglin (BD biosciences); desmin and NG2 (Millipore), and Podocalyxin (R&D Systems) [27]. Visualization of cerebral astrocytes and vascular basement membranes paraffin embedded coronal brain sections has been described previously [43]. Briefly, sections were processed for heat-based antigen retrieval at pH9 according to the manufacturer’s protocol (DAKO). Sections were then immunolabeled with anti-laminin rabbit polyclonal antibodies and anti-GFAP mAb (Millipore). Secondary antibodies conjugated with Alexa594 or Alexa488 (Invitrogen) were used to detect primary antibodies. Cell proliferation was monitored by injecting BrdUrd 2 hours before euthanasia and using Anti-BrdUrd-Alexa 594 (Invitrogen) to detect cells that had taken up this thymidine analogue. Ears were stained as previously described [44]. For analysis of staining, tissues were flat mounted and examined using a Nikon confocal A1R microscope or a Zeiss Axiovert epifluorescent microscope. Volocity software was used to generate 3D reconstructions from a z-series of 0.5 µm confocal slices. For lacZ staining, retinas were stained in X-gal solution as previously described [45]. Brain tissue was prepared by perfusion with 4% PFA prior to dissection and fixed for a further 18 hours in 4%PFA at 4°C, before being processed to paraffin and coronally sectioned.

### Q-PCR

Retinal endothelial cells were purified from collagenase digested P6 retinas using CD31-conjugated magnetic beads. RNA was prepared using the RNeasy kit (Qiagen) and cDNA was made using a high-capacity reverse transcription kit (Applied Biosystems), according to manufacturers’ protocols. Q-PCR was performed using Qiagen (SABiosciences) SYBR Green-based real time PCR Primer Assay for mouse Tgfbr2 using Quantitect primers PPM03599 and analyzed using the ΔΔC(t) method with respect to the average C(t) of 4 housekeeping genes: B2m, Rpl13a, Gapdh and Actb obtained using Quantitect primers PPM03562, PPM03694, PPM02946 and PPM02945, respectively.

### Note in Proof

While this manuscript was under review, a related paper was published (Arnold et al 2012 [46]) that included data that partially overlap with and are mainly in agreement with our findings.

## Supporting Information

Figure S1 **Endothelial Tgfbr2 expression, vascular progression and branching are significantly reduced in Tgfbr2-iKO^e^ retinas. A:** Q-PCR was used to analyse endothelial Tgfbr2 RNA expression at P6 in 3 Tgfbr2-iKO^e^ mutant and 3 control retinas. Relative expression levels (with respect to control) were calculated following normalisation to an average of 4 housekeeping genes using the ΔΔC(t) method**.** ** p<0.002. **B,C:**Progression of the retinal plexus towards the retinal periphery is significantly reduced in Tgfbr2-iKO^e^ mutants compared with controls. The ratio of the radius of the vascular plexus edge and the radius of the full retinal periphery in 13 mutants and 10 controls at P7 was calculated using the average of 3 measurements per retina as shown in C. * p<0.05. D: Vascular branching is reduced in the retinas of Tgfbr2-iKO^e^ mutants compared with controls. Branch points in the mid capillary plexus were counted in 5 fields of view for each of 3 mutants and 3 controls at P7. ** p<0.002.
(TIF)

Figure S2 **Normal phenotype of retinal astrocytes in Tgfbr2-iKO^e^ retinas.** The primary network of retinal astrocytes were examined at P6 by staining for GFAP and focussing on the region that was distal to the migrating vascular front. There were no detectable differences in the organisation of the astrocytes at the peripheral side of the migrating vascular front in the Tgfbr2-iKO^e^ mutants, compared with littermate controls. Scale bars: 50 µm.
(TIF)

Figure S3 **Tgfbr2-iKOe mutants show normal expression of the endothelial tight junction marker, Claudin 5.** Endothelial cell-cell junctions in control (A-C) and Tgfbr2-iKOe mutants (D–F) show similar levels of Claudin 5 expression**.** Claudin 5 junctions are also present in the endothelial cells of the glomerular tufts (arrows). Scale bar: 50 µm.
(TIF)

Figure S4 **Glomerular tufts in the retinas of Tgfbr2-iKO^e^ mutants are composed of disorganised aggregates of proliferating endothelial cells**. Serial sections of P14 retinas stained with H&E (A,C,E) or immunostained with anti-CD31 antibody (D,D,F) show normal small retinal capillaries on the surface of the control retina (A and black arrows, B) and clusters of multiple endothelial cells invading the neural tissue in the Tgfbr2-iKO^e^ mutants (red arrows, C–F). Panel G shows a series of confocal Z slices from a control retinal at P11 stained for BrdU and CD31. The images are ordered from the surface of the retina (left) into the neural tissue (right) and show regular capillaries (arrows) entering the neural tissue. Panel H shows a similar series of confocal images from a Tgfbr2-iKOe mutant and illustrate the proliferating endothelial cells in a glomerular tuft (inset shows digital zoom). Scale bars: 20 µm, A–F; 50 µm, G&H.
(TIF)

Figure S5 **X-gal staining is used to monitor Cre activity.** X-gal staining of a mutant (Rosa26R;Tgfbr2^fl/fl^;Cdh5(Pac)Cre^ERT2^) retina at P14 shows that Cre activation was efficient and endothelial glomerular tufts were lacZ positive (A, and inset shows two small glomerular tufts in digital zoom). The lacZ positive glomerular tufts and lack of a secondary plexus persisted at P28 (B).
(TIF)

Figure S6 **Hyaloid vasculature persists for several weeks in the Tgfbr2-iKO** ^e^ **mutants.** Retinal sections at different ages of control pups from P5 to P21 show the hyaloid microvessels (arrows in A) found between the lens (le) and the retina at P5, but are no longer present at P14. In contrast, the hyaloid vasculature of the Tgfbr2-iKO mutants persists up to 3 weeks after birth (D,E and F). Scale bar: 100 µm.
(TIF)

Figure S7 **The retinal plexus shows normal muscularisation of the arteries in Tgfbr2-iKO^e^ mutants (B,D) compared with controls (A,C).** Tgfbr2-iKO^e^ mutants show ectopic α-SMA expression in capillaries at P7 (F), which is absent in controls (E). Scale bar: 500 µm A,B; 50 µm C,D; 100 µm E,F.
(TIF)

Figure S8 **Proliferation of smooth muscle cells in the glomerular tufts of Tgfbr2-iKO^e^ retinas.** Confocal analysis following staining for BrdU and α-SMA in 6 mutant and 5 control retinas at P9 reveals double positive cells in the glomerular tufts of Tgfbr2-iKO^e^ retinas (white arrows, B) but there are no smooth muscle cells associated with the capillaries (seen in cross section in A, white arrows) in the equivalent region of the control retinas. Erythrocytes are seen as yellow cells (identified on the basis of their autofluorescence using confocal spectral unmixing) within one of the glomerular tufts in this view (blue arrow). Scale bar: 50 µm.
(TIF)

Figure S9 **The ear vasculature of the Tgfbr2-iKO^e^ mutants (B) is similar to littermate controls (A).** Note that there are no glomerular tufts in the vessels of the Tgfbr2-iKO^e^ ear. Tissue from 5 week old pups was stained for alpha smooth muscle actin (asma, red) and CD31 (green) expression and images were stitched together in the x,y dimensions using Axiovision software.
(TIF)

Movie S1 **Shows the organisation of a region of the primary and secondary retinal plexus in a control retina at P14.** Note the fine capillary plexus branching from a vein. Tissues were stained for endothelial cells (CD31, green) and smooth muscle cells (alpha smooth muscle actin, red).
(7Z) Click here for additional data file.

Movie S2 **Shows the abnormal glomerular tufts that fail to properly invade the neural tissue in the retina from a Tgfbr2-iKOe mouse at P14.** Note the smooth muscle cells associated with the glomerular tufts of endothelial cells. Tissues were stained for endothelial cells (CD31, green) and smooth muscle cells (alpha smooth muscle actin, red).
(7Z) Click here for additional data file.
