# Correction: Endothelial Expression of TGFβ Type II Receptor Is Required to Maintain Vascular Integrity during Postnatal Development of the Central Nervous System

**DOI:** 10.1371/annotation/8d859757-284b-406d-9cb9-a8776ad32fb1

**Published:** 2012-09-20

**Authors:** Kathleen R. Allinson, Hye Shin Lee, Marcus Fruttiger, Joseph McCarty, Helen M. Arthur

The fourth author's name was spelled incorrectly. The correct name is: Joseph H. McCarty. The correct citation is: Allinson KR, Lee HS, Fruttiger M, McCarty JH, Arthur HM (2012) Endothelial Expression of TGFβ Type II Receptor Is Required to Maintain Vascular Integrity during Postnatal Development of the Central Nervous System. PLoS ONE 7(6): e39336. doi:10.1371/journal.pone.0039336 

