# Supplementary figures and images for: Endothelial Expression of TGFβ Type II Receptor Is Required to Maintain Vascular Integrity during Postnatal Development of the Central Nervous System
Source: PLoS One. 2012 Jun 26;7(6):e39336. doi: 10.1371/journal.pone.0039336 (PMC3383742; doi:10.1371/journal.pone.0039336)

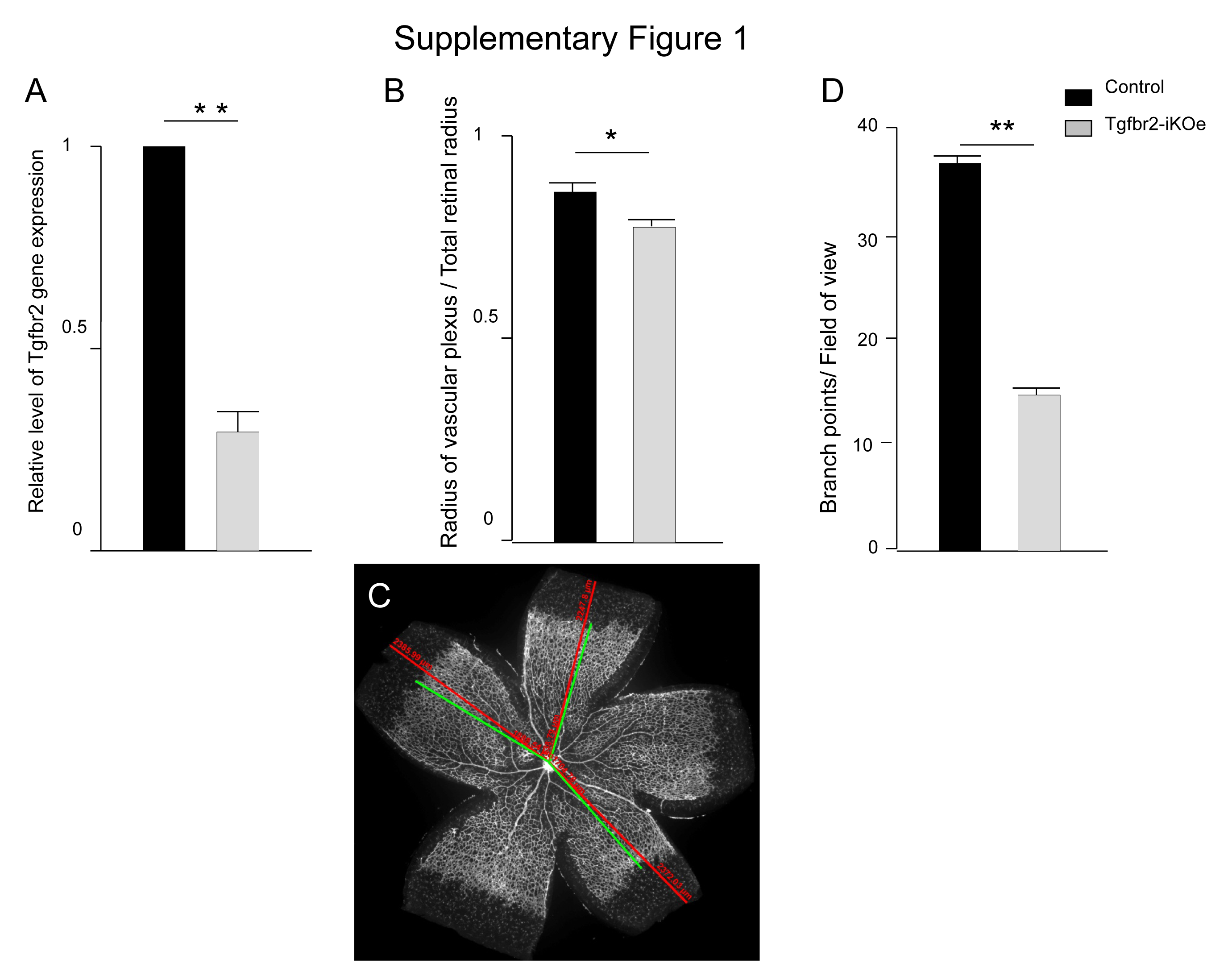

Supplement: Figure S1 — Endothelial Tgfbr2 expression, vascular progression and branching are significantly reduced in Tgfbr2-iKOe retinas. A: Q-PCR was used to analyse endothelial Tgfbr2 RNA expression at P6 in 3 Tgfbr2-iKOe mutant and 3 control retinas. Relative expression levels (with respect to control) were calculated following normalisation to an average of 4 housekeeping genes using the ΔΔC(t) method. ** p<0.002. B,C:Progression of the retinal plexus towards the retinal periphery is significantly reduced in Tgfbr2-iKOe mutants compared with controls. The ratio of the radius of the vascular plexus edge and the radius of the full retinal periphery in 13 mutants and 10 controls at P7 was calculated using the average of 3 measurements per retina as shown in C. * p<0.05. D: Vascular branching is reduced in the retinas of Tgfbr2-iKOe mutants compared with controls. Branch points in the mid capillary plexus were counted in 5 fields of view for each of 3 mutants and 3 controls at P7. ** p<0.002. (TIF) [file pone.0039336.s001.tif]

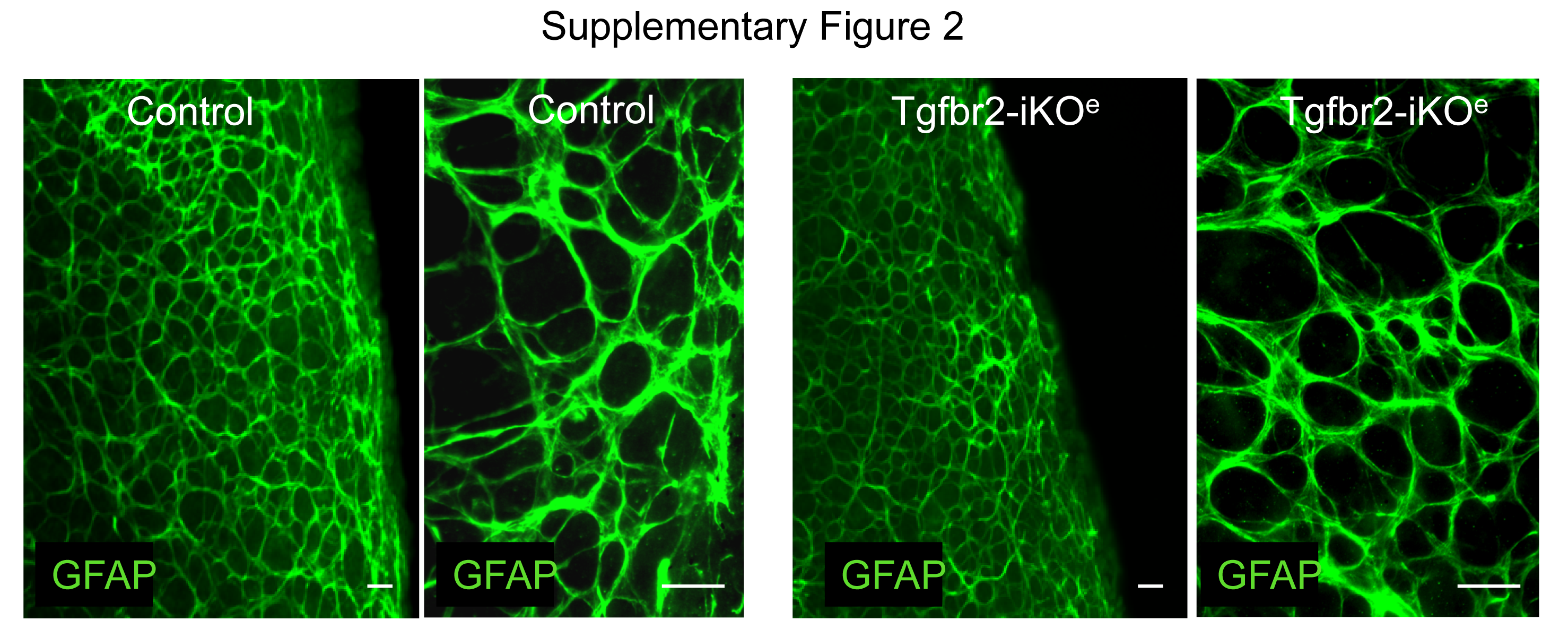

Supplement: Figure S2 — Normal phenotype of retinal astrocytes in Tgfbr2-iKOe retinas. The primary network of retinal astrocytes were examined at P6 by staining for GFAP and focussing on the region that was distal to the migrating vascular front. There were no detectable differences in the organisation of the astrocytes at the peripheral side of the migrating vascular front in the Tgfbr2-iKOe mutants, compared with littermate controls. Scale bars: 50 µm. (TIF) [file pone.0039336.s002.tif]

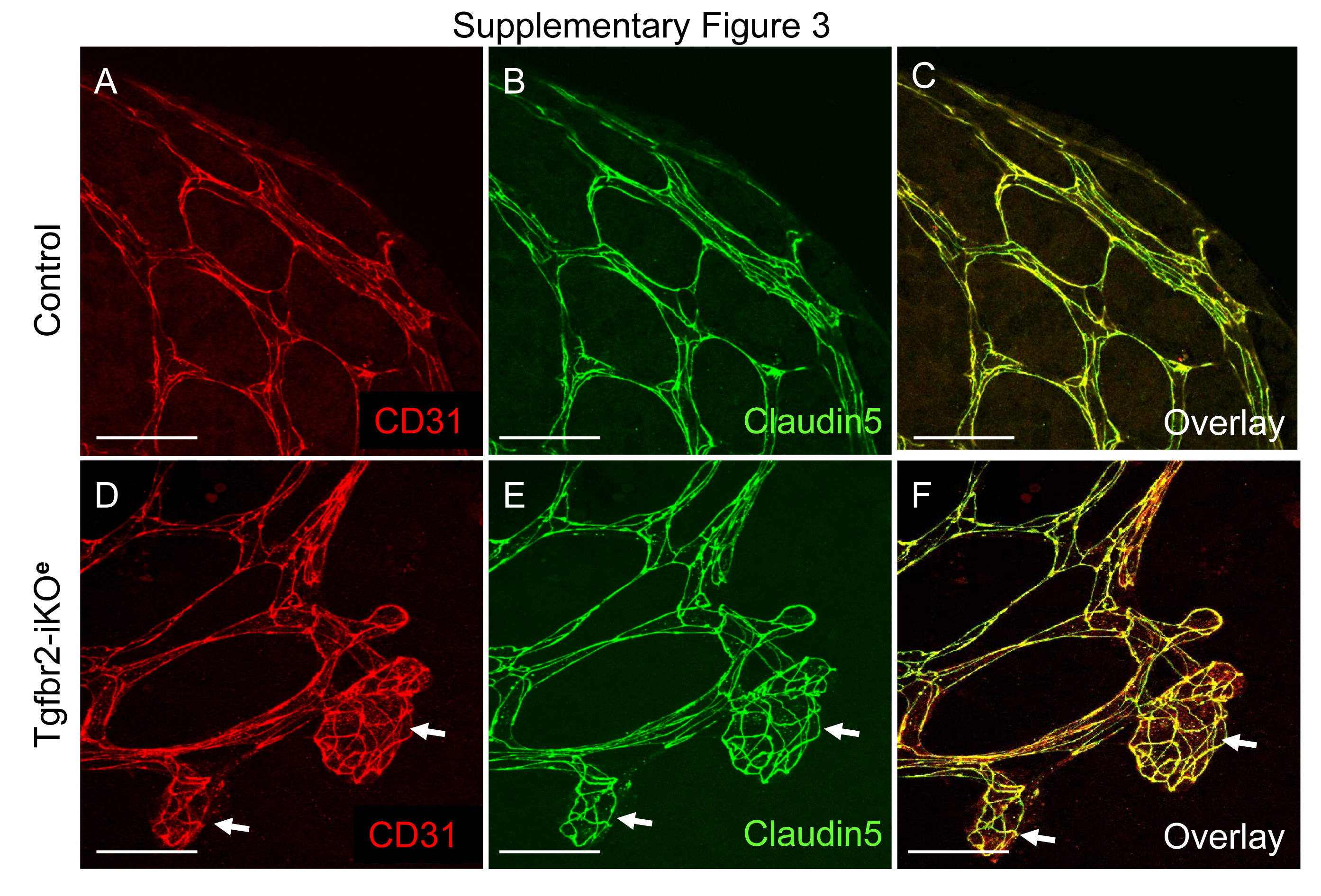

Supplement: Figure S3 — Tgfbr2-iKOe mutants show normal expression of the endothelial tight junction marker, Claudin 5. Endothelial cell-cell junctions in control (A-C) and Tgfbr2-iKOe mutants (D–F) show similar levels of Claudin 5 expression. Claudin 5 junctions are also present in the endothelial cells of the glomerular tufts (arrows). Scale bar: 50 µm. (TIF) [file pone.0039336.s003.tif]

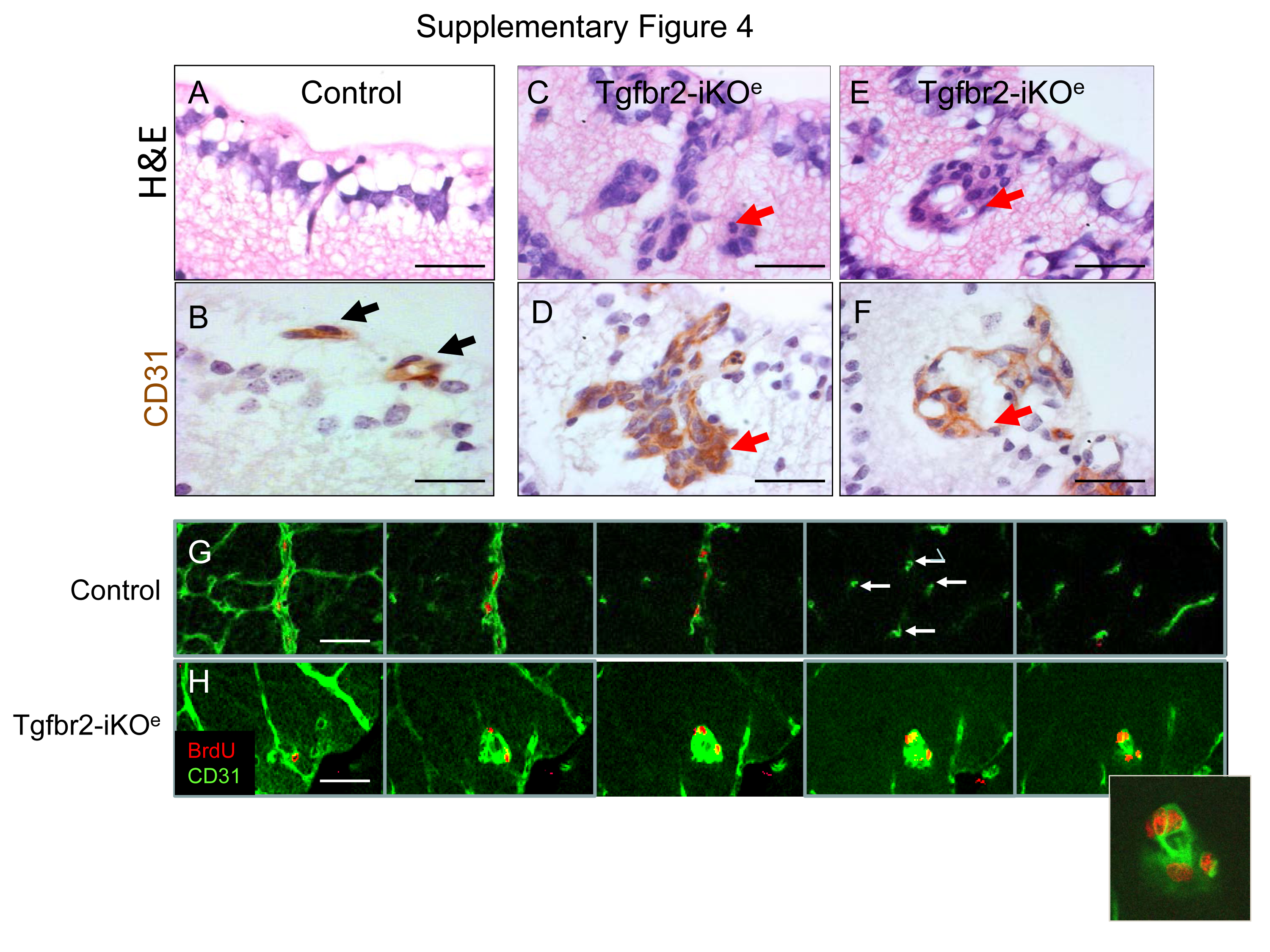

Supplement: Figure S4 — Glomerular tufts in the retinas of Tgfbr2-iKOe mutants are composed of disorganised aggregates of proliferating endothelial cells. Serial sections of P14 retinas stained with H&E (A,C,E) or immunostained with anti-CD31 antibody (D,D,F) show normal small retinal capillaries on the surface of the control retina (A and black arrows, B) and clusters of multiple endothelial cells invading the neural tissue in the Tgfbr2-iKOe mutants (red arrows, C–F). Panel G shows a series of confocal Z slices from a control retinal at P11 stained for BrdU and CD31. The images are ordered from the surface of the retina (left) into the neural tissue (right) and show regular capillaries (arrows) entering the neural tissue. Panel H shows a similar series of confocal images from a Tgfbr2-iKOe mutant and illustrate the proliferating endothelial cells in a glomerular tuft (inset shows digital zoom). Scale bars: 20 µm, A–F; 50 µm, G&H. (TIF) [file pone.0039336.s004.tif]

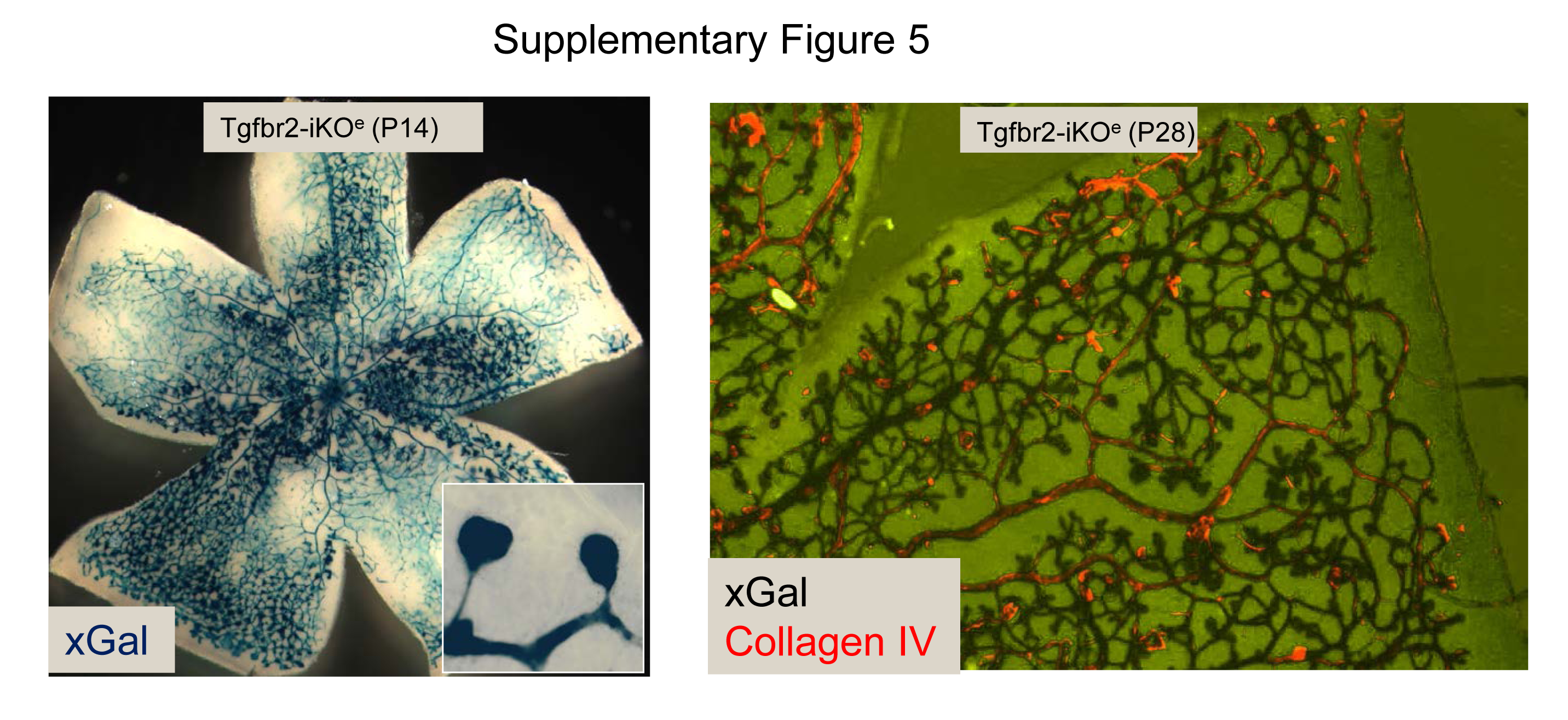

Supplement: Figure S5 — X-gal staining is used to monitor Cre activity. X-gal staining of a mutant (Rosa26R;Tgfbr2fl/fl;Cdh5(Pac)CreERT2) retina at P14 shows that Cre activation was efficient and endothelial glomerular tufts were lacZ positive (A, and inset shows two small glomerular tufts in digital zoom). The lacZ positive glomerular tufts and lack of a secondary plexus persisted at P28 (B). (TIF) [file pone.0039336.s005.tif]

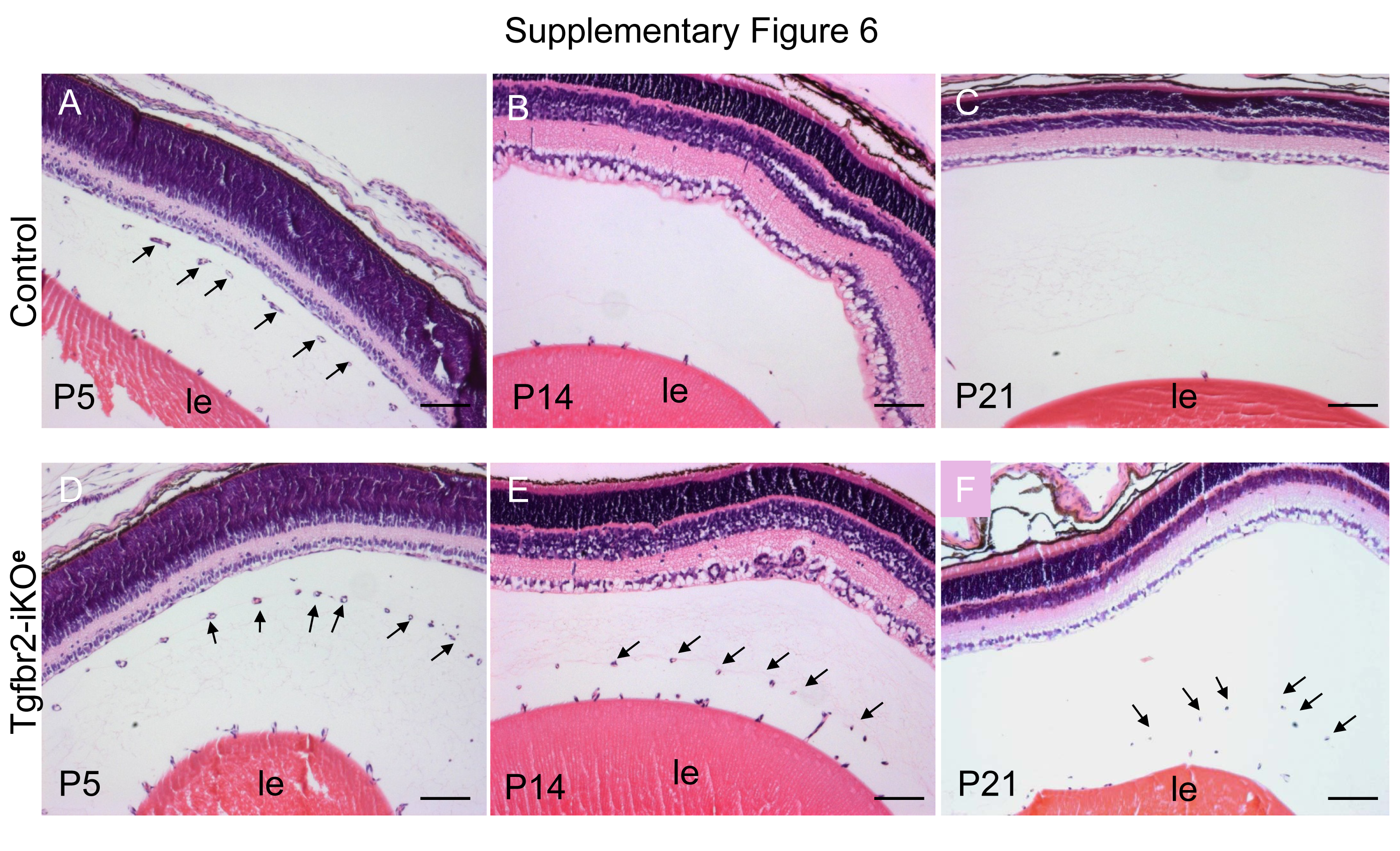

Supplement: Figure S6 — Hyaloid vasculature persists for several weeks in the Tgfbr2-iKO e mutants. Retinal sections at different ages of control pups from P5 to P21 show the hyaloid microvessels (arrows in A) found between the lens (le) and the retina at P5, but are no longer present at P14. In contrast, the hyaloid vasculature of the Tgfbr2-iKO mutants persists up to 3 weeks after birth (D,E and F). Scale bar: 100 µm. (TIF) [file pone.0039336.s006.tif]

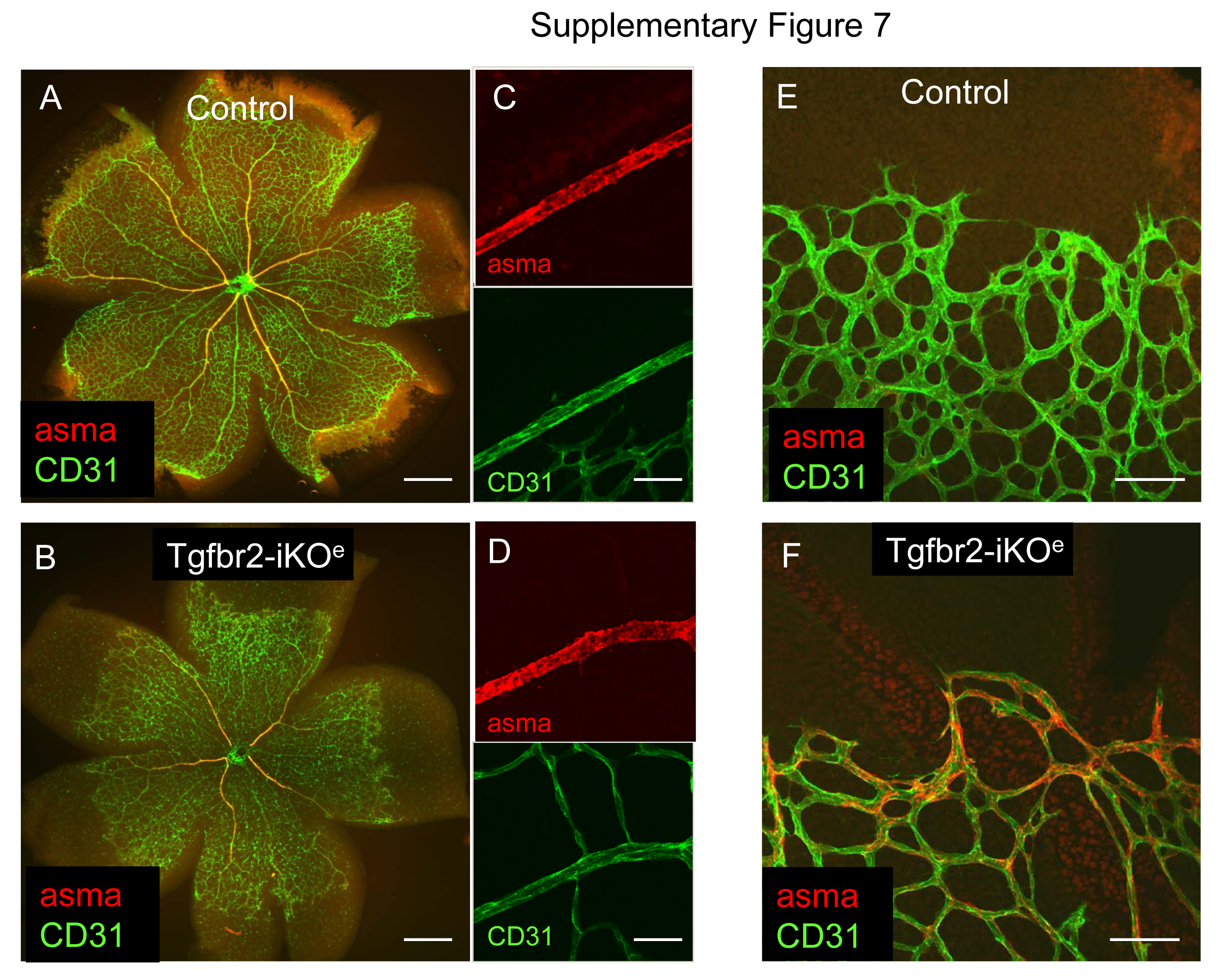

Supplement: Figure S7 — The retinal plexus shows normal muscularisation of the arteries in Tgfbr2-iKOe mutants (B,D) compared with controls (A,C). Tgfbr2-iKOe mutants show ectopic α-SMA expression in capillaries at P7 (F), which is absent in controls (E). Scale bar: 500 µm A,B; 50 µm C,D; 100 µm E,F. (TIF) [file pone.0039336.s007.tif]

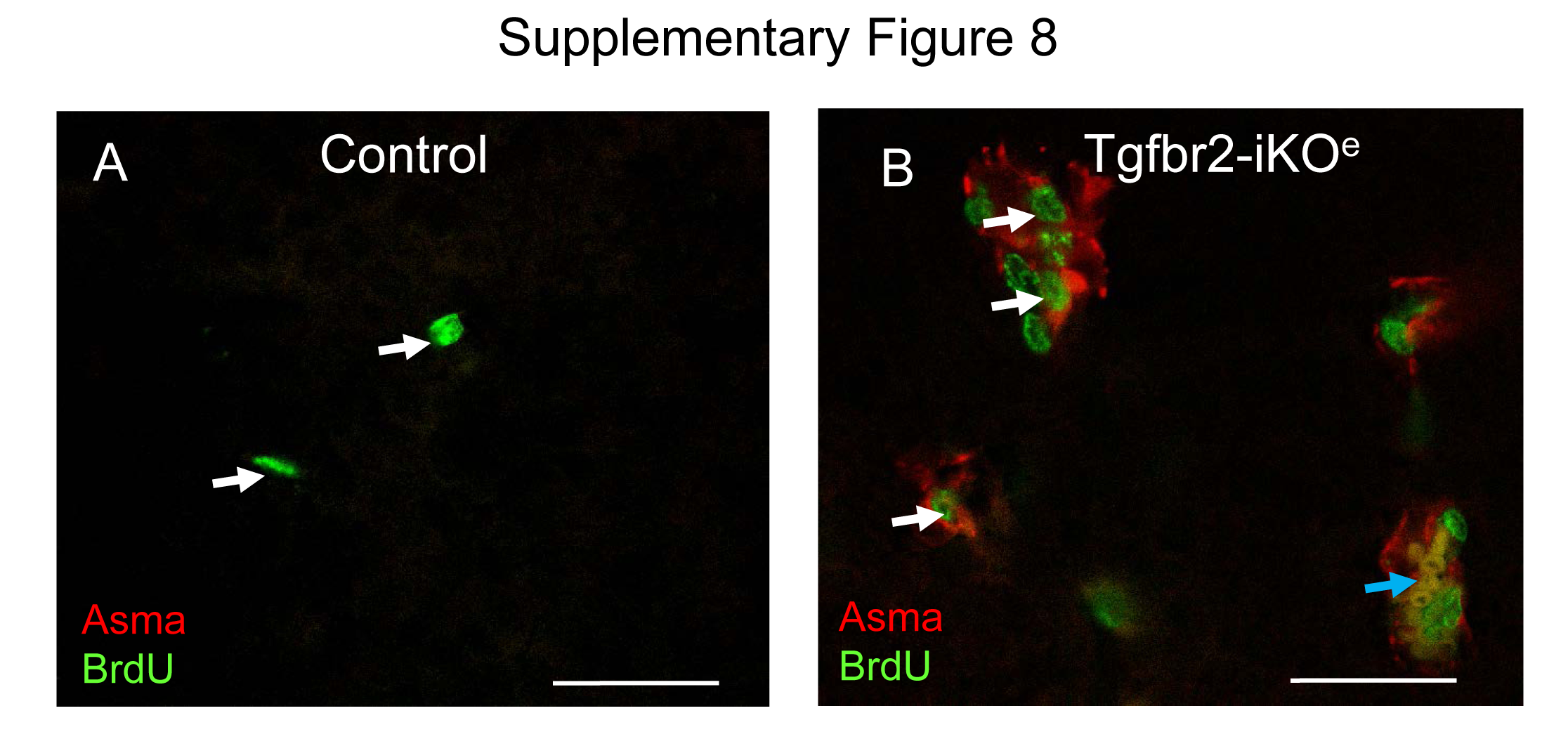

Supplement: Figure S8 — Proliferation of smooth muscle cells in the glomerular tufts of Tgfbr2-iKOe retinas. Confocal analysis following staining for BrdU and α-SMA in 6 mutant and 5 control retinas at P9 reveals double positive cells in the glomerular tufts of Tgfbr2-iKOe retinas (white arrows, B) but there are no smooth muscle cells associated with the capillaries (seen in cross section in A, white arrows) in the equivalent region of the control retinas. Erythrocytes are seen as yellow cells (identified on the basis of their autofluorescence using confocal spectral unmixing) within one of the glomerular tufts in this view (blue arrow). Scale bar: 50 µm. (TIF) [file pone.0039336.s008.tif]

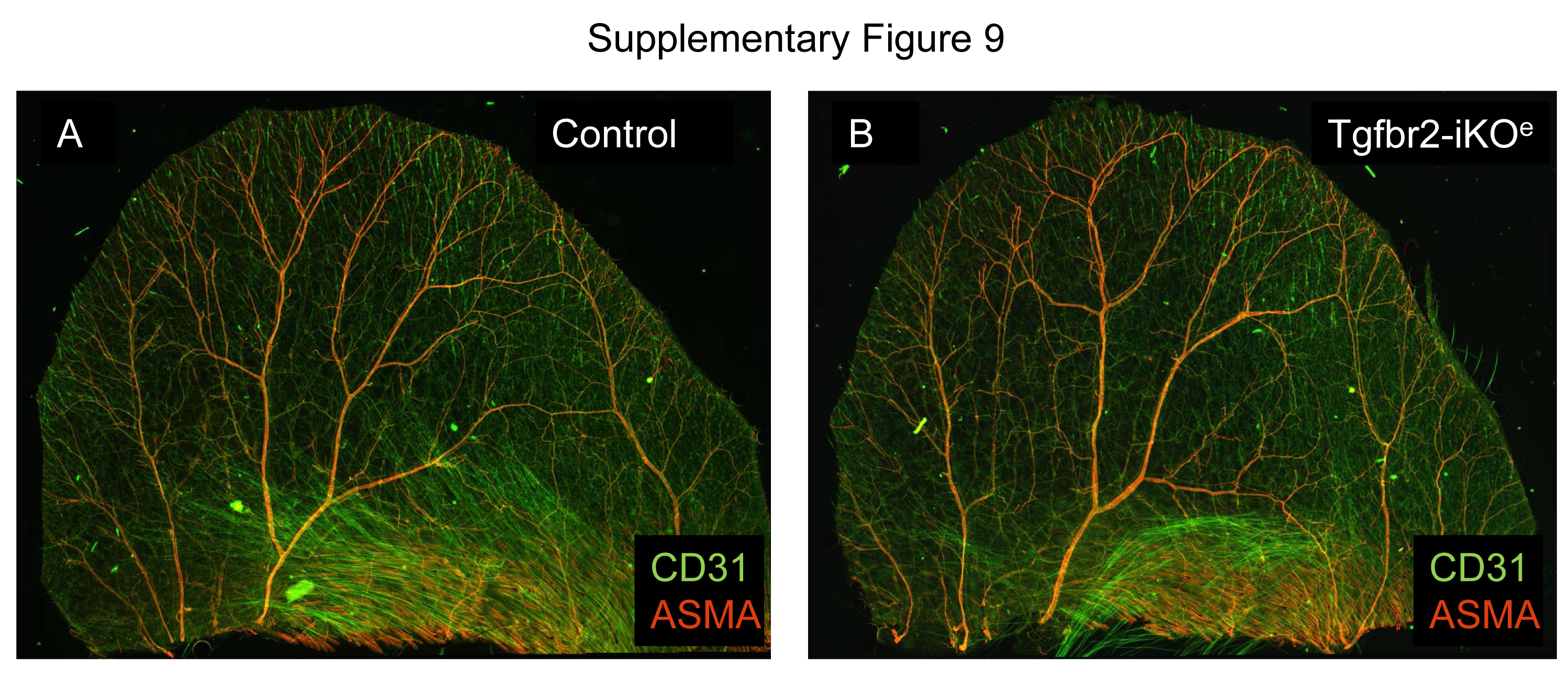

Supplement: Figure S9 — The ear vasculature of the Tgfbr2-iKOe mutants (B) is similar to littermate controls (A). Note that there are no glomerular tufts in the vessels of the Tgfbr2-iKOe ear. Tissue from 5 week old pups was stained for alpha smooth muscle actin (asma, red) and CD31 (green) expression and images were stitched together in the x,y dimensions using Axiovision software. (TIF) [file pone.0039336.s009.tif]
